# Patient Engagement with Conversational Agents in Health Applications 2016–2022: A Systematic Review and Meta-Analysis

**DOI:** 10.1007/s10916-024-02059-x

**Published:** 2024-04-10

**Authors:** Kevin E. Cevasco, Rachel E. Morrison Brown, Rediet Woldeselassie, Seth Kaplan

**Affiliations:** 1 Department of Global and Community Health, George Mason University, 4400 University Dr., Fairfax, 22030 VA USA; 2 Department of Health Administration and Policy, George Mason University, Fairfax, VA USA; 3 Department of Psychology, George Mason University, Fairfax, VA USA

**Keywords:** Meta-analysis, Systematic review, Chatbot, Conversational agents, Patient engagement, Working alliance

## Abstract

Clinicians and patients seeking electronic health applications face challenges in selecting effective solutions due to a high market failure rate. Conversational agent applications (“chatbots”) show promise in increasing healthcare user engagement by creating bonds between the applications and users. It is unclear if chatbots improve patient adherence or if past trends to include chatbots in electronic health applications were due to technology hype dynamics and competitive pressure to innovate. We conducted a systematic literature review using Preferred Reporting Items for Systematic reviews and Meta-Analyses methodology on health chatbot randomized control trials. The goal of this review was to identify if user engagement indicators are published in eHealth chatbot studies. A meta-analysis examined patient clinical trial retention of chatbot apps. The results showed no chatbot arm patient retention effect. The small number of studies suggests a need for ongoing eHealth chatbot research, especially given the claims regarding their effectiveness made outside the scientific literatures.

**Supplementary Information:**

The online version contains supplementary material available at 10.1007/s10916-024-02059-x.

## Introduction

Clinicians and patients seeking digital health (eHealth) applications face several challenges in selecting effective solutions within the $29 billion eHealth marketplace [1]. One challenge is that most eHealth applications are marketed without documented effectiveness, and few have regulatory approval [2–4]. Another challenge is health app store instability (Apple Store, Google Play), where half of mental health apps listed were removed after four months. Additionally, eHealth application development projects have a low market success rate, with only a handful of apps accounting for most application marketplace downloads [2–5]. This is complicated further as there is limited public data on how these systems benefit clinician stakeholders and high health burden populations [6].

Many eHealth applications use direct-to-consumer payment models that do not require clinical trials to validate their effectiveness [7–9]. The direct-to-consumer business model avoids costly regulatory compliance processes and effectiveness substantiation studies, but many eHealth apps still struggle to become profitable [3, 10]. Pressures to deploy applications to the market can affect business decisions and result in unrealistic timelines [11–13].

Online marketplace app attrition follows a “hype dynamics” model where a technology experiences a period of inflated expectation, followed by a trough of disillusionment, and ultimately only a few technologies achieve lasting productivity [14]. Hype dynamics can draw investment resources away from building apps using holistic design processes and can sideline critical voices within the technology community [2]. As new technologies are introduced to the eHealth applications market, industry experts and researchers must make a concerted effort to properly evaluate the performance of these tools [15].

A commercial consulting company, Gartner Inc., publishes hype cycle reports to help their clients understand the promise of emerging technologies within the context of their industry and individual appetite for risk. A 2021 Gartner report placed chatbots in their “Trough of Disillusionment” [16] where producers of the technology fail, and investments continue only if the surviving providers improve their products to the satisfaction of early adopters.

### Chatbot Patient Alliance Background

Conversational agents, or ‘chatbots’, offer a potential solution to improve eHealth app uptake and treatment adherence [17–19]. Chatbots show promise to improve end user eHealth engagement and may help users develop a therapeutic alliance with the eHealth application [17–19]. Self-guided eHealth interventions that create clinician-patient therapeutic alliance and therapeutic persuasiveness are positively correlated with real-world usage of mobile apps and web-based programs [20]. There is also evidence that applications with better design quality have better research substantiation, but this does not predict real world use and engagement [20]. Systematic reviews have shown that there are few published chatbot randomized control trials (RCT) in the eHealth market [21]; therefore, there is a small evidence base to provide guidance assessing their effectiveness [21, 22]. A newer generation artificial intelligence (AI)-based conversational large language model (LLM), such as ChatGPT, have potential to improve health care applications over traditional chatbots’ Natural Language Processing (NLP) [23]. Chatbots that improve patient eHealth adherence could expand clinical resources and mitigate clinical staff shortages.

### eHealth User Engagement Background

Improving eHealth application uptake involves developing products in a holistic framework based on participatory development approaches, persuasive design techniques, and business modeling [24]. **Randomized controlled trials** (RCTs) are the gold standard method for determining causation of treatment to outcome. RCTs treat randomly selected groups of patients with different therapies (i.e. health chatbot apps) to compare treatment effectiveness based on their health outcomes. Patients in RCTs are randomly placed into groups called trial arms. We examined two-arm RCTs because they allowed comparisons of chatbot groups to other treatments or control groups using non-chatbot treatments. Researchers can also design RCTs to collect data on an app’s user engagement. RCTs can collect data on why and when study subjects cease participation in the trial. This is referred to as loss-to-follow up (“LTFU” or “loss” or “attrition”). Loss to follow-up (LTFU) occurs when a clinical trial study subject ceases participating in a trial protocol, and we hypothesized that chatbots could improve trial retention. LTFU differs from adherence, where LTFU is defined as when participants do not return to fill in trial follow-up questionnaires. We expected an increase in chatbot user engagement would appear as a positive effect on LTFU rates because participants rarely leave a trial due to random reasons [25]. Researchers can design trials to reduce LTFU through retention techniques like offering financial incentives to participants that complete all study requirements and remain in the trial for the entire study period. Prior chatbot reviews did not compare user engagement indicators (UEI). Eysenbach’s “law of attrition” observed that in eHealth trials, a substantial proportion of users drop out before completion or stop using the application [26].

Examples of UEIs include working alliance, acceptance, and adherence. **Working alliance** measures key aspects of user engagement such as (a) agreement on the tasks of therapy, (b) agreement on the goals of therapy, and (c) development of an affective bond [27]. **Acceptance** loss is a measure of user satisfaction and subject loss due to dissatisfaction, and is referred to as disenchantment/discontinuance rejection in clinical trials [26]. **Adherence** loss is where subjects stop using the chatbot during the trial and is called non-usage attrition in clinical trials [26].

Based on prior systematic reviews, we did not expect to find sufficient data in literature review studies to quantitatively assess chatbot UEI effects [28–30]. However, there was an opportunity to examine chatbot effects on RCT participant retention [25, 31]. The goal of the meta-analysis was to provide a chatbot UEI assessment using qualitative and quantitative study subject attrition results from eHealth RCT chatbot studies. We compared chatbot clinical trial arms against controls with human interventions, non-chatbot/non-human interventions, and assessment only. We expected chatbot intervention arms to show similar LTFU rates as non-chatbot control arms; because in peer-reviewed health research LTFU was normally similar across trial arms [32].

## Methods

### Study Context

Our approach was to perform a systematic review and meta-analyses on user engagement indicators (UEIs) that measured health domain specific studies through trial participant retention and loss to follow-up. The systematic review qualitatively examined UEIs: working alliance, acceptance, and adherence. App adoption was not assessed because it was assumed that subjects in the trial used the chatbot once they had gone through trial recruitment, selection, and informed consent processes. Software monitoring tools and system logs enabled measuring trial subject UEIs for eHealth products more thoroughly than RCTs for medication trials, but eHealth UEIs were not collected consistently [29].

We used a random effects meta-analysis to compare LTFU attrition rates between intervention and control groups. Meta-analysis methods provide an independent, outside view of eHealth product effectiveness and can mitigate hype and product planning cognitive biases. Also, meta-analysis methods statistically combine and analyze data from separate studies, therefore they play a central role in synthesizing research findings, especially in fields where studies were typically onerous or expensive (e.g., clinical trials) [33].

Clinical trial study designers set a predetermined minimum target of people to recruit into a trial to achieve statistical inference. Failure to both recruit and retain sufficient participants can affect study quality and bias the results. Other factors have influenced LTFU in mobile app trials showing lower attrition rates associated with acceptance-based interventions, participant monetary compensation, younger age, and employing engagement reminders [34, 35]. Chatbot study subject attrition was an important indicator because it may be a source of bias and systematic error leading to an incorrect estimation of association between exposure and outcome [36].

This study was limited to randomized control trials because the study design provided quantitative comparison of chatbot intervention groups to control groups. Chatbot eHealth meta-analyses to-date have focused on specific health conditions resulting in few studies focusing on comparing primary health effects [37, 38]. For example, a meta-analysis of mental health chatbots yielded just four studies with comparable primary health effects [37].

### Literature Review Methodology

During March 2022, we searched PubMed, ProQuest and EBSCO CINAHL electronic databases to identify studies with chatbot trials for review. The PubMed search engine queried most of the content in MEDLINE and PubMed Central (PMC). This study followed Preferred Reporting Items for Systematic reviews and Meta-Analyses (PRISMA) review methodology, but the protocol was not registered [39]. Additionally, two chatbot systematic reviews were identified with lists of trials referenced [28, 38]. Search terms are located in Appendix A. The prevalence of published chatbot studies increased dramatically starting in 2016 following chatbot cloud platform software releases by large technology companies [40]. Therefore, queries were limited to articles published between January 1, 2016, and February 28, 2022.

The following definition of chatbot was used for inclusion decisions: “A chatbot is a computer program which responds like an intelligent entity when conversed with. The conversation may be through text or voice. Any chatbot program that understands one or more human languages by Natural Language Processing” [41].

There were several inclusion/exclusion criteria that were used to select studies. The chatbot application must have been patient-facing with a health education, monitoring, or treatment related mission. Study primary outcome measures were about the user’s health outcome versus a chatbot’s system or algorithmic performance, user’s system interaction, or user’s chatbot design preferences. Chatbots with primary outcome supporting clinical trial infrastructure were excluded (e.g., data collections, patient interaction). This study excluded chatbots intended for clinicians, health workers, and researchers, as well as studies that used communication modalities that did not provide user conversational interaction (IVR, one-way text messaging).

Selected articles included those with chatbot users enrolled in experimental designs: trials, experiments, randomized controlled trials (RCTs) with pre-post evaluation design. Studies must have had both a chatbot trial arm and a control arm for comparison and included only studies with an adult population age 18 and over. All studies were included in the synthesis with subgroup analysis by control arm intervention type (non-human/non chatbot, human, assessment only). Excluded were systematic reviews (systematic, scoping, meta-analyses) and conference summaries.

The inclusion/exclusion criteria were intended to identify studies with RCTs supporting effectiveness claims specific to the patient or consumer market. Results included studies published in English. The study’s primary outcome measures were focused on the user’s health outcome. Chatbots with primary outcome supporting clinical trial infrastructure, such as data collections and patient interaction research, were excluded.

Three researchers independently searched the three databases (PubMed, ProQuest, EBSCO CINAHL) using the database search engines. HubMeta software identified duplicate studies which were screened by one reviewer to be discarded. Two reviewers independently screened all database search result record titles and abstracts. Articles resulting from the title and abstract screening were full text screened independently by two reviewers and conflicting decisions were resolved by a third reviewer; any inconsistencies were discussed. A single reviewer assessed a list of articles included in two systematic reviews [37, 38], and results were discussed with a second reviewer to be considered for inclusion. Duplicate records from the systematic reviews were excluded.

The reviewers developed a standardized form to extract data which included data element description and data coding format instructions (Appendix B). Data coded by the two reviewers were compared, with discrepancies being resolved through discussion.

A quality assessment of each report was carried out to determine if the study followed the Consolidated Standards of Reporting Trials (CONSORT) criteria [42]. All studies were evaluated against the CONSORT checklist. No study bias assessment of the search results was conducted because many of the bias tools focus on quality of the primary outcome while this systematic review and meta-analysis was focused on secondary UEIs and loss-to-follow-up administrative data. This review collected data associated with conversational agent app trial recruitment and retention, provided descriptive results, and assessed chatbot versus control arm UEIs in the synthesis.

### Data Analysis Methods

To characterize the studies, we captured information about the trial environment. Countries where the trials were conducted were coded because there was a lack of literature on between-country differences in how eHealth trials handle multinational chatbot design, UEI, or attrition. Also, because women are historically under-represented in clinical trials, the proportion of female subjects was coded [35]. Study UEI data was collected for Working alliance, Acceptance, Adherence, and end user survey instruments. This included system usage information frequently used to measure subject interaction with system through system logs, subject interaction counts, and engagement duration.

The metafor package in R was used to perform effect size estimate calculations and display forest plots and publication bias analyses [33]. Differences between chatbot and control arm LTFU rates were assessed for heterogeneity using metafor with models fit using maximum likelihood estimators. Results were described with Cochran Q, I^2^ index, and Tau across all studies and by control arm subgroup. Causes of heterogeneity were explored with intervention type subgroups and moderators.

We used log relative risk as the effect size measure by comparing the chatbot arm retention against total participants of the chatbot and control. Moderators included payment of incentives to study subjects and mean study subject age. Incentives were typically negatively associated with study attrition, and older subjects were less likely to adopt technology. Studies that reported statistically significant results or clinically relevant results were published more often and can lead to publication bias [43]. Additionally, trials that had poor initial outcomes can be discontinued before completion; this can lead to further publication bias. To test for publication bias related to standardized adherence, the Egger test was applied. Results were adjusted after randomization for loss for study eligibility, study errors or other non-participant attrition to determine if loss can be attributed to trial arms.

## Results

### Study Selection

**Fig. 1:**
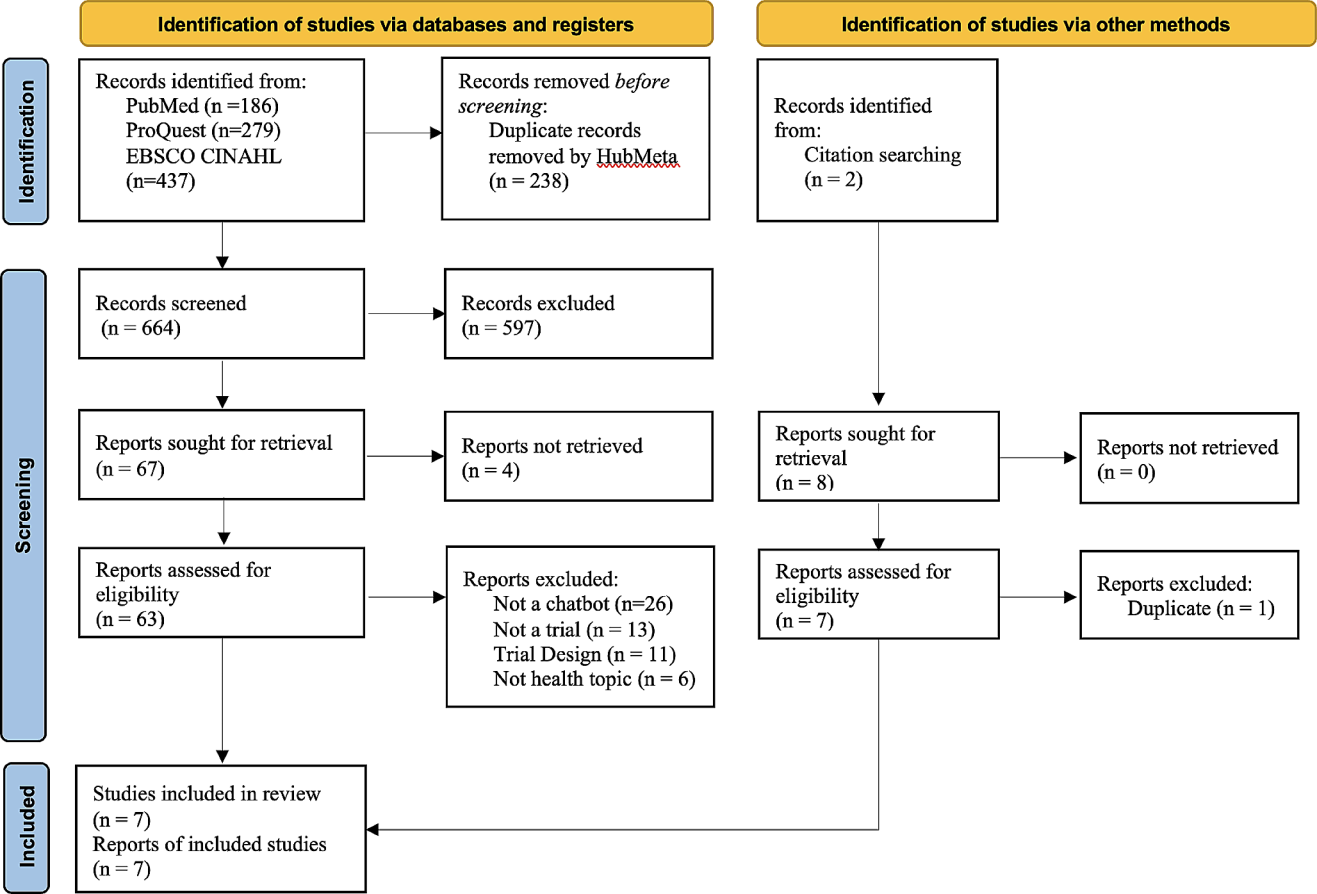
Summary of article selection process [39]

The search resulted in 14 articles, seven from the database searches and seven from citation searches. After 238 duplicates were removed, 664 articles were screened from which 67 full text documents were reviewed (Fig. 1). The most frequent reason an article was excluded during full text review was that the intervention did not meet the definition of a chatbot (*n* = 26). Twenty-four studies were not controlled trials with comparable arms (*n* = 13) or lacked detailed data on all trial arms (*n* = 11). Studies were also excluded that did not examine health topics, or the primary outcome was about chatbot system performance or chatbot user experience (*n* = 6). Inter-rater reliability was high due to specific screening criteria requiring multi-arm, randomized trial design. Kappa statistic for reports assessed for eligibility was 95%.

### Study Characteristics

Chatbot intervention arms were compared against four human interventions, four assessment-only controls without intervention, and six controls with non-conversational interventions such as static medical education materials or text messaging reminders. The final list of selected articles is provided in Table 1. We found eight behavioral health applications and six in other clinical domains. There were seven applications that addressed mental health conditions such as depression and anxiety.

In terms of gender composition, the average study population was 65.8% female. A prior study found health and wellbeing smartphone apps were more frequently downloaded by women than men [44] and all mental health articles had higher female than male participation. With regard to country, Burton was the only multinational study. The app’s virtual agent was an animated human-based character whose gender, clothing style, voice, and language were selectable during configuration.

There were eight apps that provided care without clinician participation in the intervention, four where the app interacted with the patient to support ongoing clinical care, and one that provided education (Table 1). An example of a study that provided care was Gong, with personalized diabetes support, monitoring, and motivational coaching via an embodied conversational agent, through a series of modules covering blood glucose monitoring, healthy eating, physical activity, medication taking, and foot care. An example of a study that supported care was Burton, which recruited subjects through mental health clinicians’ active caseloads. The intervention included elements of cognitive behavioral treatments to guide self-reflection and provide summaries of progress for the patient and their supervising clinician, with an overview of changes in the patient’s conditions.

### Quality Assessment

Most studies followed CONSORT publishing guidelines where nine (64.3%) declared CONSORT compliance or providing a checklist. The CONSORT quality review identified a lack of reporting of harms or unintended effects in twelve studies (85.7%), an important patient safety omission in mental health treatment applications. Randomization was reported but with few details on mechanism and concealment of groupings. Blinding of subjects and caregivers was performed in one study (7.1%), and three studies blinded the data assessors (21.4%). The lack of blinding may be common where subjects are part of ongoing mental health therapy or when pharmacotherapy blinding designs may not translate to eHealth [45]. Primary and secondary outcomes were clearly defined, but many outcomes did not use validated measures (Table 2).

The “Subjects Randomized” column indicates the number of subjects ultimately included in the trial. Statistical power calculations and resulting study subject recruitment goals were not found for five of the studies. Of the nine studies with recruitment targets, only five recruited sufficient subjects (35.7%). Results included one pilot study (Anan) with no power target, and one study (Sandoval) that ended early due to funding and logistical issues.

**Table 1:** Characteristics of the studies included in the meta-analysis

Author	Country	Health Condition	Power Target (N)	Subjects Randomized (N)	Female (N%)	Duration (mo)
Hauser-Ulrich, 2020 [46]	Switzerland	Pain care support	115	102	80.4	2
Pot, 2017 [47]	Netherlands	HPV Vaccine education	1200	9124	100.0	3
Anan, 2021 [48]	Japan	Neck, shoulder, back pain care	N/A	121	18.2	3
Jack, 2020 [49]	USA	Preconception care	353	528	100.0	12
Gong, 2020 [50]	Australia	Diabetes care	697	187	41.7	12
Burton, 2016 [51]	Scotland, Romania, Spain	Depression care support	52	28	66.7	1
Echeazarra, 2021 [52]	Spain	Blood pressure care support	N/A	112	42.0	24
So, 2020 [53]	Japan	Problem Gambling care	198	254	19.8	1
Greer, 2019 [54]	USA	Mental health post cancer care	N/A	45	80.0	1
Berger, 2017 [55]	Online	Anxiety disorder care	176	139	98.0	2
Fitzpatrick, 2017 [56]	USA	Anxiety and depression care	70	70	81.0	1
Sandoval, 2017 [57]	USA	Depression care	N/A	45	62.2	2
Zwerenz, 2017 [58]	Germany	Depression care	128	229	60.7	3
Fulmer, 2018 [59]	USA	Anxiety and depression care support	N/A	75	71.2	1

The examination of working alliance, acceptance, and adherence to assess technology hype-dynamics showed few quantitatively comparable results. Only the Zwerenz study compared both working alliance and acceptance across trial arms. Hans-Ulrich only compared working alliance between study arms, and the Fulmer study provided a descriptive comparison of intervention and control arms. There is little comparable quantitative data for health chatbots UEIs to provide an accurate outside view to counter eHealth product planning cognitive biases and technology hype.

Two studies examined **working alliance**. The Hauser-Ulrich working alliance survey indicated chatbot intervention group participants reported significantly higher bond scale values at the follow-up measure compared to those reported at the baseline measure. Non-interactive control group bond score declined. Zwerenz reported improving the patient-app bond, but intervention to control study randomization had different baselines that may have introduced bias in these working alliance results. For **acceptance**, all but one study reported customer satisfaction quantitative results for chatbot trial arms. Most of the quantitative results were based on custom surveys, with multiple metrics, that are not comparable across studies. Normalizing **adherence** results across studies is difficult and the results did not report attrition proportions at different points in time during a trial to illustrate if attrition curves are comparable.

Hauser-Ulrich and Zwerenz studies captured working alliance measures using validated instruments (Working Alliance Inventory-Short Revised (WAI-SR) and Helping Alliance Questionnaire (HAQ)) [27, 60]. Hauser-Ulrich implemented a smartphone cognitive behavior therapy (CBT) text-based health care chatbot intervention for pain self-management in patients with ongoing or cyclic pain. The study compared working alliance between control group and intervention group subjects. The chatbot based app intervention provided a coach with a drawn image of a face that acted as a guide through the CBT lesson materials. The coach also instructed participants on how to integrate mindfulness into their daily routine and provided users with a relaxation exercise. The control group received motivational messages with a quotation every week, which only involved content unrelated to chronic pain. Results from the bond scale of the WAI-SR indicate chatbot intervention group participants reported significantly higher bond scale values at the follow-up measure compared to those reported at the baseline measure (*p* = 0.005) (µ (SD): preintervention 5.43 (1.27), postintervention 5.89 (1.1)). Control group bond score decreased during the study period with wider variance in responses (µ (SD): preintervention 5.58 (1.44), postintervention 4.51 (2.08)). These results indicated a desire of participants to interact with a chatbot in the same way they do with humans. This study did not achieve the declared study subject recruiting target.

Both intervention and control groups in the Zwerenz study first received inpatient psychodynamic psychotherapy that consisted of individual and group psychotherapy, creative psychotherapy interventions, and adjunct treatments like patient education and physical training. However, intervention and control groups received different follow-up support. The intervention group received 12 weeks of access to an interactive, online, internet-based self-help program. The control group were granted access to a non-interactive online platform providing 12 weekly modules with specific topics regarding depression. Most of the control group content was taken from the patient version of the German medical guidelines. The authors stated that the higher discharge HAQ scores suggest a desire of participants to interact with a chatbot in the same way they do with humans, and supports the theory of media equation, which claims that people tend to treat computers or other media as if they were real people [58]. However, the randomization process that allocated patients to treatment and control groups resulted in different baselines potentially biasing the working alliance post-treatment result.

Table 2 is a summary of acceptance and adherence UEIs. Only two studies compared acceptance measures between the chatbot arm and controls. The Sondoval, So, Jack and Pot study control arms were assessment-only with no intervention. Zwerenz study compared acceptance at the end of the intervention with 79% of intervention group subjects reporting they were “quite” or “very” satisfied and the control group at 46%. A Chi-squared analysis showed the chatbot acceptance was statistically higher in the human intervention control group (χ2 = 25.98; *p* < 0.001; d = 0.74). The study’s positive findings on acceptance, retention, and depression outcome support the potential benefit of chatbot based therapeutic modalities.

The Fulmer chatbot was an adjunct to therapy which delivered interventions rooted in a variety of psychological modalities such as CBT, mindfulness-based therapy, emotionally focused therapy, acceptance and commitment therapy, motivational interviewing, self-compassion therapy, and interpersonal psychotherapy. The Fulmer study reported descriptive results comparing trial arm acceptance where 86% (43/50) subjects reported being overall satisfied with the app and only 60% (14/24) in the control intervention reporting such. Designed to supplement the role of a trained therapist, the study offered evidence that chatbots can serve as cost-effective and accessible therapeutic agents.

Raw adherence attrition proportions at different points in time during a trial can illustrate attrition curves and allow between trial arm and between study comparisons [26]. However, quantitative adherence results over time and chatbot to control arm comparison results were not reported. The Berger and Sandoval studies included evidence-based instruments for end user acceptance using Client Satisfaction Questionnaire (CSQ-8) [61] and System Usability Scale (SUS) [62]. The Hauser-Ulrich and So studies measured acceptance through net promoter score (NPS) [63]. Anan and Gong did not use surveys to gather user input on UEIs. The remaining studies used custom acceptance surveys with and without evidence supporting survey question development. Adherence measures are a mix of system and chatbot usage logs and end user surveys. The Pot, Gong, Burton, So, Greer, Berger, and Zwerenz studies reported duration usage to assess if users engaged throughout the study period. The Pot, Anan, and Berger studies reported other results that were not declared in the study methods section.

**Table 2:** Measures of acceptance and adherence user engagement

Study	Working Alliance Measures	Acceptance Measures	Adherence Measures			Survey Instruments
			System logs	Usage Count	Usage Time	
Hauser-Ulrich, 2020 [46]	Yes	Usefulness, easy to use, enjoyment, recommend to others	Yes	Yes	No	WAI-SR, NPS
Pot, 2017 [47]	No	Web site and virtual assistant experience	Yes	Yes	Yes	Custom
Anan, 2021 [48]	No	No	Yes	Yes	No	None
Jack, 2020 [49]	No	Satisfaction	Yes	Yes	No	Custom
Gong, 2020 [50]	No	No	Yes	Yes	Yes	None
Burton, 2016 [51]	No	Recommend to others, virtual agent experience	Yes	Yes	Yes	Custom
Echeazarra, 2021 [52]	No	Easy to use	No	No	No	Custom
So, 2020 [53]	No	Recommend to others	Yes	Yes	Yes	NPS
Greer, 2019 [54]	No	Helpful, Recommend to others	Yes	Yes	Yes	Custom
Berger, 2017 [55]	No	Customer satisfaction	Yes	Yes	Yes	CSQ-8
Fitzpatrick, 2017 [56]	No	Customer satisfaction, Experience	Yes	Yes	No	Custom
Sandoval, 2017 [57]	No	Ease of use	No	No	No	SUS
Zwerenz, 2017 [58]	Yes	Customer satisfaction	No	Yes	Yes	HAQ
Fulmer, 2018 [59]	No	Customer satisfaction	Yes	Yes	No	Custom

### Results of Syntheses

**Fig. 2:**
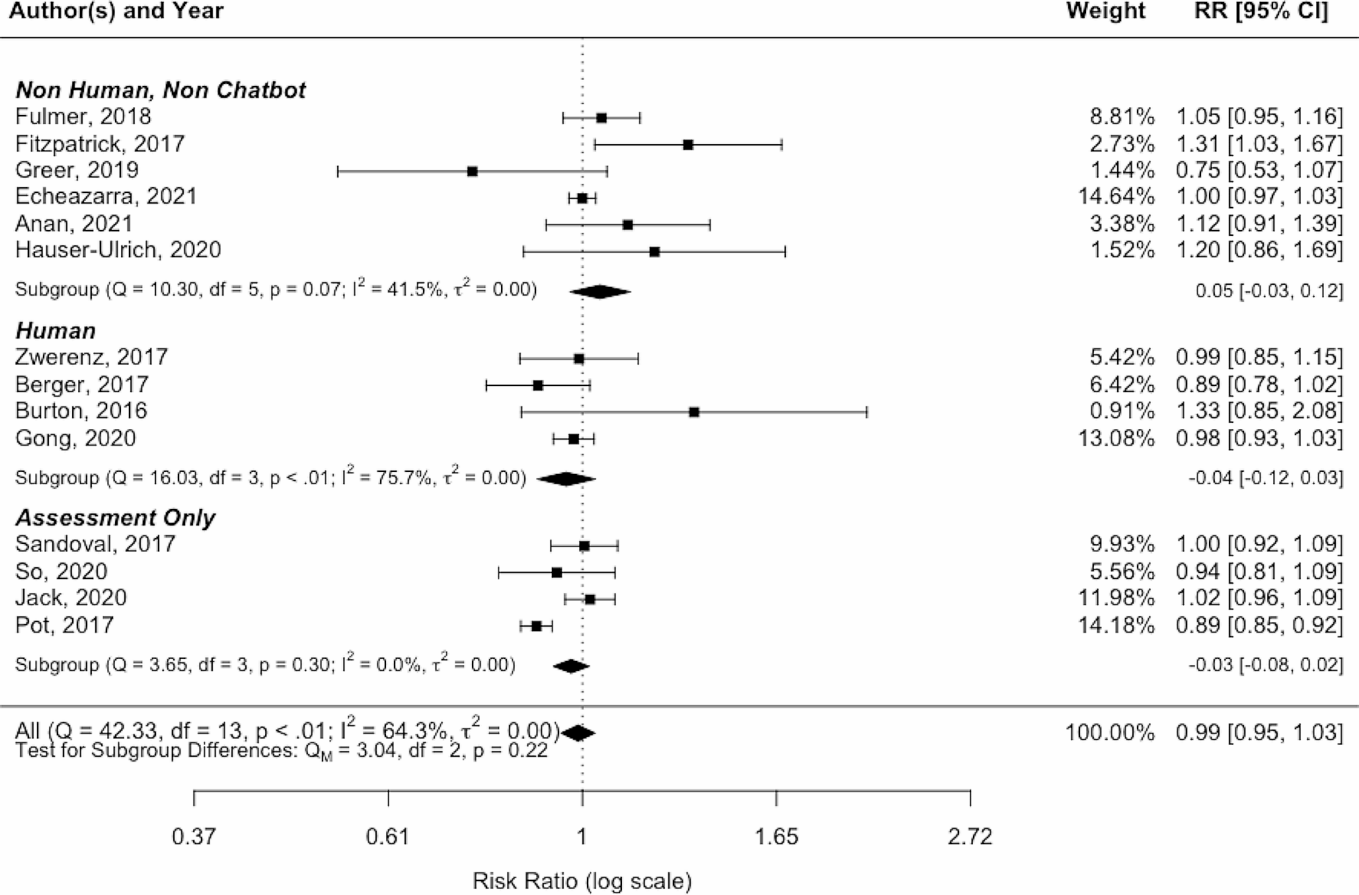
Forest plot comparing chatbot arm loss to follow-up with three types of control arms. Higher study subject trial retention has a higher risk ratio. The risk ratio is shown on a log scale indicated at the bottom of the figure

Meta-analysis of all 14 studies produced an overall effect size of 0.99 (95% CI 0.95–1.03; *p* < 0.01), which indicated no statistically significant difference between chatbot loss to follow-up and three other types of intervention. The results of the meta-analysis are shown in Fig. 2. Studies with a horizontal line (Fig. 2) that spans Risk Ratio of “1” are inferred to have a chatbot trial LTFU that is statistically equivalent to the non-chatbot arm. The horizontal diamonds in all three groups also span Risk Ratio line, indicating no statistical difference as a group between chatbot LTFU and non-LTFU arms. Heterogeneity was relatively high, with 64.3% of the variation of the risk ratio explained by the between-study heterogeneity. The test of homogeneity of study-specific effect sizes was rejected (Q = 42.33, df = 13, *p* < 0.01) in the test for subgroup differences.

Non-human, non-chatbot comparison subgroup had a moderate level of heterogeneity with chatbot arms. The analysis showed 41.5% (I^2^) of the total variability in loss to follow-up was due to true heterogeneity of between-studies variability. The confidence interval point estimate was small (RR 0.05) indicating little clinical difference, where chatbot arms showed slightly higher study retention than control arms. The human to chatbot comparison subgroup had a high level of heterogeneity (I^2^ = 75.7%) where chatbot arms had slightly lower study subject retention. The non-intervention, assessment only subgroup was very homogenous (I^2^ = 0.0%) with slightly lower chatbot study retention.

Because we could not reject our hypothesis that chatbot and non-chatbot trial arms had the same LTFU based on the omnibus test (Q_M_ = 3.25, df = 3, *p* > 0.05), we applied moderators to the model to assess their influence on loss-to-follow-up. Most heterogeneity was explained by study differences in age and incentives, not by chatbot enabled interventions. Adding age and incentives paid to participants as moderators resulted in an I^2^ unaccounted variability of 0.04% and a statistically significant Q for moderators (QM = 26.0555, df = 2, *p* < 0.001). Both age (est 0.01, *p* < 0.0001) and incentives (est 0.17, *p* < 0.0001) were statistically significant.

Only the Fitzpatrick study showed a statistically significant difference with higher chatbot study arm retention (RR 1.31, 95%CI: 1.03, 1.67). The results support the study’s findings that the mental health app was highly engaging and more acceptable than the information control group intervention. The study with the largest number of subjects (Pot et al.) was the only result with a statistically significantly lower retention than the chatbot arm (RR 0.89, 95%CI: 0.85, 0.92). The study used an intention to treat method with imputed missing values. There were higher dropout rates in subgroups typically associated with lower vaccination rates (immigrants, lower levels of education, lower disease risk perception, and lower self-efficacy). The control arm was assessment-only, and the study did not report if the attrition was due to the intervention design or due to vaccination hesitant subjects self-selecting out of a vaccination education intervention.

Study LTFU was commonly reported as failure to submit study surveys but did not adjust for application usage cessation during the trials. Detailed trial arm subject enrollment, LTFU, and moderators are available in Appendix D.

Only the Fitzpatrick RCT study showed significantly higher chatbot retention over the control arm and our result is supported by an observational study funded by the application’s owner (Woebot) [64]. Woebot was shown to have working alliance scores comparable to those in previously published studies comparing chatbots to human-delivered services across different treatment modalities [64] and was working towards independent evaluation for FDA clearance. No regulatory clearance/approval had been granted at the time of this meta-analysis.

When examining LTFU between chatbot and control arms, this meta-analysis found no statistically significant difference, which matches general findings on LTFU in clinical trials [32]. The remaining heterogeneity between studies may be explained by participant incentives and age. These results were similar regardless of control arm type. This is similar to other findings on chatbot arm clinical interventions that found a lack of evidence that chatbot primary effect is clinically important [37]. The LTFU effects suggest that including chatbot technology in eHealth applications is not a substitute for holistic eHealth product development framework using participatory development approaches, persuasive design techniques, and business modeling.

### Reporting Biases

The Egger’s regression test was conducted to determine if there is a relationship between the observed effect sizes and LTFU. The results of this test showed that there was potential for asymmetry, and indicated potential publication bias (beta = -0.08; 95% CI: -0.15, -0.01; *p* = 0.01). More details about publication bias and the funnel plot are available in Appendix E.

## Discussion

Chatbot based apps offer the potential to help clinicians and patients using eHealth applications improve uptake and treatment adherence. This systematic review of UEIs demonstrated that chatbot RCT studies did not generally compare chatbot arm working alliance, acceptance, and adherence measures to control arms. The LTFU meta-analysis showed no statistically significant difference between loss to follow-up between chatbot interventions and control arms. Therefore, chatbots arms did not have better trial subject retention than non-chatbot arms.

The practical and research implications of our findings are expanded upon below, then discussion of the study limitations.

### Practical Implications

Perhaps the foremost conclusion from this review is that assessment of chatbots for these outcomes is very seldom undertaken. The few studies that are conducted frequently do not recruit sufficient subjects to substantiate their study aims. Our expansive search yielded only fourteen RCTs using health chatbot technology globally for eight behavioral health and six other clinical domains. This resulted in few comparisons of chatbot enabled applications with other treatment options. This is a small count when examining the $29B eHealth marketplace.

The funnel plot and Egger analysis indicate there may be publication bias where trials with poor results were not published. Furthermore, the current findings imply that chatbot developers may be hesitant to share any results they do gather.

Collectively, the lack of available results noted above, paired with the potential for publication bias, suggests that the real impact of chatbot enabled applications are largely unknown. The results matched prior studies showing there is a small evidence base to provide guidance assessing eHealth effectiveness. As such, practitioners should be wary to recommend chatbot apps without regulatory compliance and effectiveness substantiation studies. The American Psychiatric Association offers an App Evaluation Model (AEM) that can help clinicians and patients choose mental health applications that include consideration of clinical foundations of effectiveness claims.

### Clinical Implications

Health systems commonly require eHealth software to be approved through a governance board process before deployment into patient care settings [65]. Our results showed there was little RCT data to support a chatbot app navigating the governance process due to lack of effectiveness evidence, and patient safety information. Payors require RCTs evidence to approve reimbursement which is necessary for the health system’s return-on-investment business case [66]. Health systems may instead implement chatbots and LLMs for non-patient-facing use cases that can be successfully defended in the eHealth governance process with internally available information. (e.g. documentation management, summarizing literature) [67].

### Research Implications

With regard to research implications, this review led to several takeaways. First, LTFU should not be viewed only as a risk to statistical power. Per Eyesnbach’s Law of Attrition, dropout rates are high in eHealth trials, and UEI metrics as determinants of attrition should be highlighted, measured, analyzed, and discussed. Few studies were found that used evidence-based instruments for UEI analysis. Information about the study subject attrition rate and at what point subjects leave a trial can provide app design feedback. Given the app market failure rate, application development and business audiences should leverage clinical trials to measure UEIs, regardless of regulatory or insurance payment implications.

Given the dearth of studies noted above, researchers and app developers should be encouraged to submit their findings regardless of results. There are business reasons for not publishing unsuccessful eHealth app trials. Journal editors could encourage such submissions by publishing only UEI effects. They could do so by devoting special issues to this topic, for example, and by encouraging pre-registration and the submission of null findings.

Adherence research in the pharmaceutical market has been underfunded, but companies are financially successful through insurance reimbursement despite 50% of patients not adhering to their prescribed medication regimens [68]. Conversely, low eHealth app uptake and adherence has stranded many direct-to-consumer eHealth companies between incurring product development costs and achieving sustainable revenue streams. The study results suggest that using chatbot end user interfaces may not overcome the low user engagement driving poor eHealth financial outcomes. Technology companies have been successful in video game uptake, to the extent that internet gaming disorder is stressed in the Statistical Manual of Mental Disorders (DSM-5) [69]. LLMs could ethically, effectively, and equitably improve uptake and adherence through personalized communication, simplifying complex information, interactive engagement, and emotional support. LLMs have already been trained on patient-doctor dialogue datasets, and future research could examine how these LLMs can improve patient facing UEIs in addition to replicating doctor-patient interactions [67, 70].

However, current eHealth clinical trial design and practice does not provide the basis to determine if these new conversational Artificial Intelligence models would improve working alliance, acceptance, and adherence. Given the potential and rapid advancements of LLMs, it is important to explore integration methods into clinical trials that take into account supervised implementation, cost considerations, and ethical oversight. Although LLMs have the potential for improving personalized medicine by increasing health literacy and providing easily available and understandable health information, there is risk to users if the systems are not carefully designed and evaluated. The applications may lack context to provide the LLMs effective information about specific cases [71]. For example, in mental health applications, LLMs may provide generic responses and may not be able to account for all the complex factors that can impact mental health [72].

### Limitations

This review has several limitations worth noting. First, the available amount of effect sizes means any interpretations about results must be made with caution. Also, as noted above, there was significant heterogeneity among these 14 effect sizes, thus calling for even greater caution when interpreting these findings. In addition, the studies covered a several year-period, during which technological innovations in chatbot technology were made. The degree to which the importance of particular chatbot technology, and chatbot features in general, improves adherence remains an open question. This study did not explore the potential impact of technology addiction nor digital literacy effects on UEIs. This study does not include studies that examine UEIs outside of multi-arm randomized trials, such as those specifically looking at working alliances, or non multi-arm clinical trials. This review does not include unpublished trials.

## Conclusion

This approach to indirectly capture UEIs through loss-to-follow-up rates showed no chatbot arm effect, and the heterogeneity between chatbot and other arms are explained by trial incentives and age. Most studies did not use validated instruments to capture the UEIs necessary to determine if chatbots improve end-user working alliance, acceptance, and adherence. We identified a bias where studies with poor loss to follow-up may not have been published. High eHealth product failure rates, and poor end user effectiveness could be mitigated by capturing better UEI data that feeds back into clinicians and eHealth product development teams to counter biases and hype.

## Electronic Supplementary Material

Below is the link to the electronic supplementary material.

Supplementary Material 1

## Data Availability

Data supporting the findings of this study are available within the paper and supplemental file. Individual study trial arm participation counts, loss to follow-up counts, age, and incentives are available in Appendix D. Reasonable requests for other data can be addressed to the corresponding author.
