# Supplementary material for: Patient Engagement with Conversational Agents in Health Applications 2016–2022: A Systematic Review and Meta-Analysis
Source: J Med Syst. 2024 Apr 10;48(1):40. doi: 10.1007/s10916-024-02059-x (PMC11004048; doi:10.1007/s10916-024-02059-x)

**Patient engagement with conversational agents in health applications 2016-2022: A Systematic Review and Meta-Analysis**

Kevin E. Cevasco^1^, Rachel E. Morrison^1^, Rediet Woldeselassie^2^, Seth Kaplan^3^

Affiliations

^1^George Mason University, Department of Global and Community Health, Fairfax, VA

^2^George Mason University, Department of Health Administration and Policy, Fairfax, VA

^3^George Mason University, Department of Psychology, Fairfax, VA

**Corresponding Author**

Correspondence to Kevin Cevasco at [kcevasco@gmu.edu](mailto:kcevasco@gmu.edu)

Kevin Cevasco ORCID 0000-0002-7082-4836

# Appendix A

Systematic Review Search

Chatbot search terms are based on previous chatbot review search terms (Milne-Ives et al., 2020; Safi, Abd-Alrazaq, Khalifa, & Househ, 2020) and use Boolean operators.

Search string:

(Speech recognition software or "Conversational agent*" or "embodied conversational agent*" or chatbot* or avatar* or "dialog* system" or "speech recognition software" or "voice recognition software" or "virtual assistan*" or "virtual nurs*" or "virtual patient" or "virtual coach*" or "virtual agent" or "relation* agent" or "assistance technol*" or "intelligent assistan*" or "digital assistan*" or "natural language interface" or "interactive computer agent" or "computer-assisted instruction" or "natural language communication" or "natural language understanding" or "unconstrained natural language processing")

AND ( Tele* OR Telemedicine OR Tele-medicine OR Telehealth OR Tele-health OR Telecare OR Mobile Health OR mHealth OR Electronic health OR eHealth)

AND (web OR applicati* OR technology OR software)

Jan 1, 2016 - Feb 28, 2022

Search results were converted to RIS format, a standardized tag format developed by Research Information Systems, then imported into HubMeta for further review.

This search combined and expanded upon several prior systematic review search strategies that did not take advantage of standardized terminologies. We have updated supplemental file with examples of MeSH terms that could be used in future searches:

- Telemedicine: MeSH Unique ID: D017216
- Natural Language Processing: MeSH Unique ID: D009323
- Speech Recognition Software: MeSH Unique ID: D049250
- Avatar: MeSH Unique ID: D000095902

# Appendix B

Main study table data structure:

- ID: Each record was assigned an identification number (N)
- Health Condition: Health related condition addressed by the chatbot, (e.g. diabetes, depression, etc.) (Text)
- Number of arms in the trial design (N)
- Does the study provide financial or other incentives for trial enrollment and/or retention? (Y/N)
- Percentage of subjects that are female (N%)
- Average age across trial arms (N)
- Study design (Text)
- Working alliance measures (Text)
- Acceptance measures (Text)
- Adherence measures (Text)
- Subject enrollment power target (N)
- Subjects randomized (N)

Trial arm table data structure:

- Trial arm type: (Categorical: Control, chatbot)
- Intervention type: (Categorical: human, chatbot, non-human/non-chatbot, assessment only). Human control arms involve human interaction with a health worker or clinician. Examples are standard of care where ethics require clinical setting or remote treatment. Non-human/non-chatbot control arms involved non conversational technology like web pages or non-interactive text messaging. Assessment only are non-intervention controls where subjects are only surveyed.
- Arm size (N): Number of subjects randomized into this trial arm.
  - Loss to follow-up due to administrative reasons not attributed to user decision to continue with a trial will be removed from arm size and loss to follow-up results: (N)
- Loss to follow-up (N): Number lost to follow-up at last subject data collection point study arms have in common.
- Chatbot engagement or adherence measure accounted for in loss to follow-up: (Y/N)
- Duration: Months study conducted (N in months)

Data coding was recorded in two tables due to variations within study between chatbot and control arms. The main table recorded study-wide information with a second table with a row for each trial arm. The main study and trial arms are linked by a study identification number.

# Appendix C

## Coding guide

| Data element | Instructions | Description |
| --- | --- | --- |
| Main table |  |  |
| ID | (starts at 100) | Meta analysis study identification number. Assigned after full text review |
| DOI | Imported from Zotero | Study DOI identifier |
| Coder | “ | Initials of person that coded the data |
| Title | “ | Study published title |
| Publication Title | “ | Journal or other publication title |
| Issue | “ | “ |
| Volume | “ | “ |
| Date | “ | Date published |
| Author | “ | Study authors |
| Health Condition | Text  (e.g. diabetes, depression, etc.) | Health related condition addressed by the app |
| Purpose | Options:  Treatment, monitoring, patient education, other) | What is goal of intervention |
| Arms (#) | (N) | Number of arms in the trial design.  (E.g. control, chatbot intervention, other intervention) |
| Enrollment Power Target | (N),  if no study power calculation, then code as “N/A” | Number of subjects to be recruited based on a power calculation. |
| Randomized subjects | (N) | How many subjects were cleared through to the randomization phase? |
| Country | Text  (if online study without a stated country, then code as “online”) | Country where study was conducted |
| Incentives? | (Y/N) | Does the study provide financial or other incentives for trail enrollment and/or retention? |
| Female % | (N%) | Percentage of subjects that are female |
| Age (mean) | (N) | Mean age of the study population. If only provided for each arm then estimate study-wide mean with proportion |
| Age (sd) | (N) | Standard deviation of the population age |
| Trial Duration (months) | (N) | If not in months, round weeks or days to nearest number of months |
| Data Collection Start | MM/DD/YYYY or MM/YYYY | Date the first data collection started |
| Data Collection End | MM/DD/YYYY or MM/YYYY | Date of final data collection |
| Trial arm table |  |  |
| ID |  | Corresponding ID from main table |
| Arm type | Control, chatbot |  |
| Primary outcome | Text | Description of primary measure we are using to code this arm. Do not code secondary measures. |
| Intervention type | Options: “Human”,” Chatbot”, “Non-human/Non-chatbot”, “Assessment only” | Mode the study subject interaction. Assessment only means no intervention in control arm. |
| Setting | Options: home, research/clinical, other | Where is the subject when interacting with the trial? |
| Platform | Options:  mobile, computer browser software, special device.  “N/A” for non-technology arms like paper document or human interaction | Technology platform the subject users to interact with the chatbot, control, or alternative intervention. |
| Reminders | (Y/N) | Does the trial provide reminders to the subject to engage in the intervention? |
| Arm Name | Text | Name of the trial arm as |
| Arm N | (N) | Number of subjects randomized into this trial arm. Adjust down randomized baseline for loss for eligibility, study errors or other non-participant attrition |
| Loss to follow-up | (N) | Number lost to follow-up at end of study. |
| Engaged? | (Y/N) | Chatbot engagement or adherence measure accounted for in loss to follow-up: |
| Admin Loss | (N) | Loss to follow-up due to administrative reasons not attributed to user decision to continue with a trial. Will be removed from arm size and loss to follow-up results: (N) |
| Working alliance measure | Text | Validated instrument to measure working alliance between patient and chatbot app |
| Acceptance measure | Text | Measures for customer satisfaction, user experience |
| Adherence measure | Text | Measures for frequency and duration of chatbot app use |

# Appendix D

## Results of individual studies

Trial arm details with trial arm retention, loss to follow-up frequencies, and moderator variables

| Author | Control arm type | Chat Retain | Chat Loss | Control Retain | Control Loss | Age (mean) | Incentive |
| --- | --- | --- | --- | --- | --- | --- | --- |
| Hauser-Ulrich, 2020 | Non-human/non-chatbot | 38 | 21 | 23 | 20 | 43.8 | No |
| Pot, 2017 | Assessment only | 2197 | 2358 | 2481 | 2088 | 43.7 | Yes |
| Anan, 2021 | Non-human/non-chatbot | 48 | 13 | 42 | 18 | 42.1 | No |
| Jack, 2020 | Assessment only | 232 | 30 | 231 | 35 | 27.4 | Yes |
| Gong, 2020 | Human | 89 | 4 | 92 | 2 | 57.0 | No |
| Burton, 2016 | Human | 12 | 2 | 9 | 5 | 38.7 | No |
| Echeazarra, 2021 | Non-human/non-chatbot | 55 | 0 | 57 | 0 | 52.1 | No |
| So, 2020 | Assessment only | 87 | 36 | 99 | 32 | 37.3 | Yes |
| Greer, 2019 | Non-human/non-chatbot | 16 | 9 | 17 | 3 | 25.0 | Yes |
| Berger, 2017 | Human | 57 | 13 | 63 | 6 | 42.0 | No |
| Fitzpatrick, 2017 | Non-human/non-chatbot | 31 | 3 | 25 | 11 | 22.2 | Yes |
| Sandoval, 2017 | Assessment only | 25 | 0 | 20 | 0 | 28.7 | Yes |
| Zwerenz, 2017 | Human | 85 | 30 | 85 | 29 | 48.0 | No |
| Fulmer, 2018 | Non-human/non-chatbot | 50 | 0 | 24 | 1 | 22.9 | Yes |

# Appendix E

The publication bias results are presented in Figure E1 using the trim-and-fill method to create a funnel plot, which shows the distribution of studies on the log risk scale along with corresponding p-values and measures the sensitivity of the applied literature review. The trim and fill method filled in 3 corresponding studies that created a more symmetric distribution to prevent any extreme results. This funnel plot was created using the random-effects model without moderators.

***Fig E1****: Contour-enhanced funnel plot using the trim and fill method to estimate number of studies missing from meta-analysis*


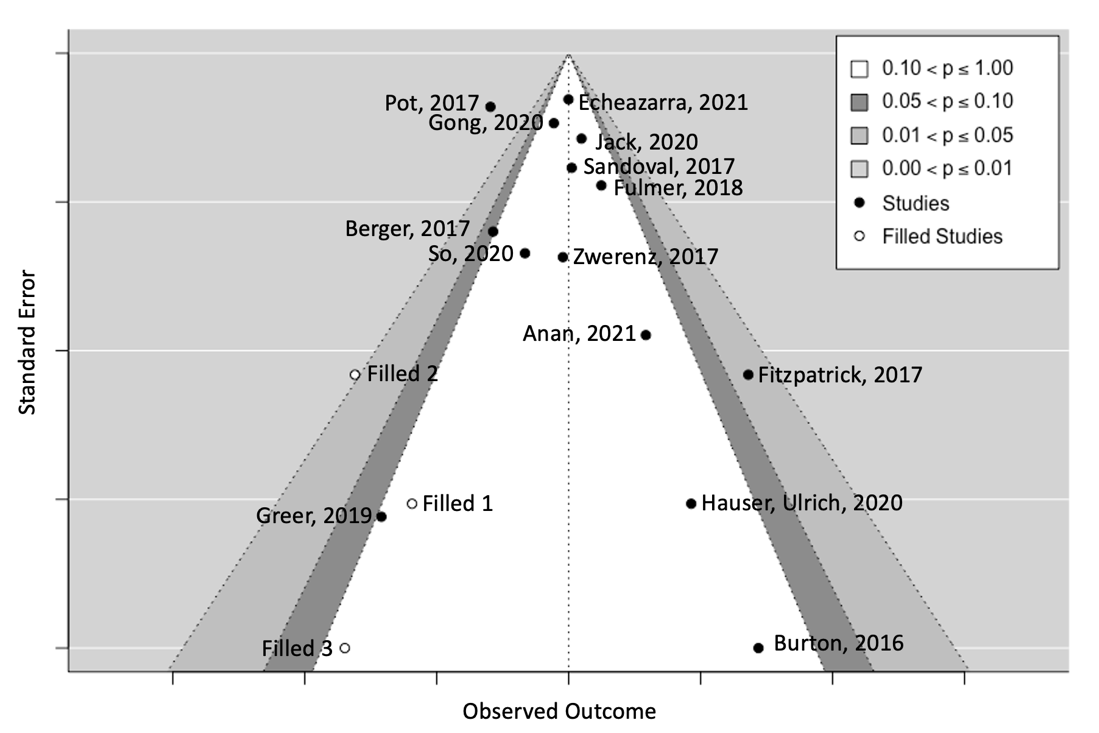

Supplement: Supplementary file 1 — Supplementary Material 1 [file 10916_2024_2059_MOESM1_ESM.docx]
